# A Localized Complex of Two Protein Oligomers Controls the Orientation of Cell Polarity

**DOI:** 10.1128/mBio.02238-16

**Published:** 2017-02-28

**Authors:** Adam M. Perez, Thomas H. Mann, Keren Lasker, Daniel G. Ahrens, Michael R. Eckart, Lucy Shapiro

**Affiliations:** a Department of Developmental Biology, Stanford University School of Medicine, Stanford, California, USA; b Department of Biology, Stanford University, Stanford, California, USA; c Department of Biochemistry, Stanford University School of Medicine, Stanford, California, USA; d Stanford Protein and Nucleic Acid Facility, Stanford University School of Medicine, Stanford, California, USA; University of California—Berkeley; California Institute of Technology/HHMI

## Abstract

Signaling hubs at bacterial cell poles establish cell polarity in the absence of membrane-bound compartments. In the asymmetrically dividing bacterium *Caulobacter crescentus*, cell polarity stems from the cell cycle-regulated localization and turnover of signaling protein complexes in these hubs, and yet the mechanisms that establish the identity of the two cell poles have not been established. Here, we recapitulate the tripartite assembly of a cell fate signaling complex that forms during the G_1_-S transition. Using *in vivo* and *in vitro* analyses of dynamic polar protein complex formation, we show that a polymeric cell polarity protein, SpmX, serves as a direct bridge between the PopZ polymeric network and the cell fate-directing DivJ histidine kinase. We demonstrate the direct binding between these three proteins and show that a polar microdomain spontaneously assembles when the three proteins are coexpressed heterologously in an *Escherichia coli* test system. The relative copy numbers of these proteins are essential for complex formation, as overexpression of SpmX in *Caulobacter* reorganizes the polarity of the cell, generating ectopic cell poles containing PopZ and DivJ. Hierarchical formation of higher-order SpmX oligomers nucleates new PopZ microdomain assemblies at the incipient lateral cell poles, driving localized outgrowth. By comparison to self-assembling protein networks and polar cell growth mechanisms in other bacterial species, we suggest that the cooligomeric PopZ-SpmX protein complex in *Caulobacter* illustrates a paradigm for coupling cell cycle progression to the controlled geometry of cell pole establishment.

## IMPORTANCE

Lacking internal membrane-bound compartments, bacteria achieve subcellular organization by establishing self-assembling protein-based microdomains. The asymmetrically dividing bacterium *Caulobacter crescentus* uses one such microdomain to link cell cycle progression to morphogenesis, but the mechanism for the generation of this microdomain has remained unclear. Here, we demonstrate that the ordered assembly of this microdomain occurs via the polymeric network protein PopZ directly recruiting the polarity factor SpmX, which then recruits the histidine kinase DivJ to the developing cell pole. Further, we find that overexpression of the bridge protein SpmX in *Caulobacter* disrupts this ordered assembly, generating ectopic cell poles containing both PopZ and DivJ. Together, PopZ and SpmX assemble into a cooligomeric network that forms the basis for a polar microdomain that coordinates bacterial cell polarity.

## INTRODUCTION

Cellular polarity underlies diverse biological events, including cell differentiation. The asymmetrically dividing bacterium *Caulobacter crescentus* is a model system for single-cell polarity, as every cell division produces two daughter cells that differ in their morphology, replication competency, and size (1). Prior to cytokinesis in *Caulobacter*, distinct sets of signaling proteins localize to opposite cell poles where they dictate the cell fate of the nascent daughter cells.

**FIG 1:**
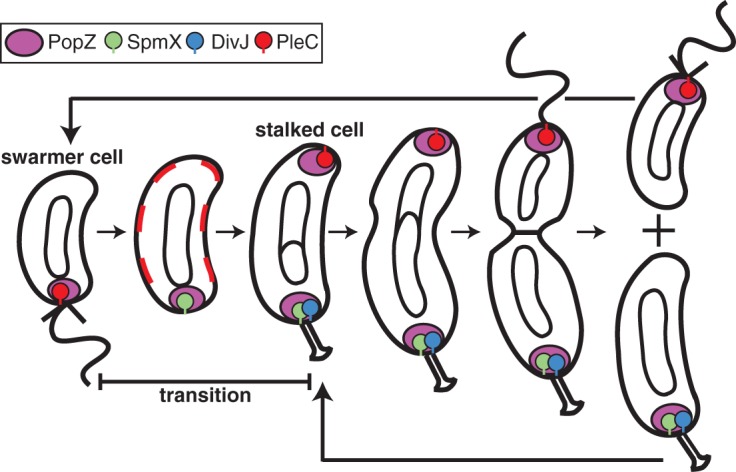
Polar complex transitions during *Caulobacter* cell cycle. One pole of the swarmer cell bears a single flagellum and a space-filling matrix composed of the polymeric protein PopZ. The membrane-bound PleC phosphatase, which promotes the swarmer cell fate, is positioned at this flagellum-bearing pole. As the swarmer cell differentiates into a stalked cell, the flagellum filament is released and PleC is dispersed around the cell membrane. Concurrently, the SpmX protein is synthesized and colocalized with PopZ, and the flagellum is ejected. Following SpmX localization, the DivJ kinase localizes at the cell pole, and stalk biogenesis and DNA replication initiate. Following the initiation of replication, a second PopZ matrix assembles at the pole opposite the stalk, and PleC reaccumulates at this incipient swarmer pole. Sequestration of PleC and DivJ at opposite poles promotes the swarmer and stalked cell fates, respectively, of the daughter cells. Sticks on protein cartoons represent transmembrane tethers. The circles and theta structures within the cell outline represent the replication of the swarmer cell’s single chromosome, and the wavy and straight lines at the cell pole represent the flagellum and pili, respectively.

One of the progeny, the sessile stalked cell, immediately enters S phase and initiates chromosome replication. The other progeny, the motile swarmer cell, enters G_1_ phase and is incapable of DNA replication. The swarmer cell undergoes a period of differentiation to become a stalked cell, culminating in the generation of further progeny (Fig. 1). During this G_1_-S transition, the developing swarmer cell releases the PleC phosphatase from the flagellated cell pole, sheds its polar flagellum, begins biogenesis of the polar stalk appendage, and initiates DNA replication. Synthesis of the DivJ histidine kinase marks the end of the G_1_-S transition, enabling the initiation of chromosome replication and driving the stalked cell genetic program. Newly synthesized DivJ is positioned at the nascent stalked pole, where it remains localized to propagate the stalked cell fate throughout future divisions (2–4).

Localization of DivJ to the nascent stalked pole depends on several factors. A microdomain composed of the PopZ polymeric network, which marks the flagellated pole in swarmer cells (5, 6), is necessary for the polar localization of DivJ in addition to many other cell fate factors that localize to the cell poles (6, 7). One PopZ-dependent factor, SpmX, colocalizes with PopZ immediately upon synthesis at the beginning of the G_1_-S transition (8–11). SpmX is necessary for the stalked pole localization and activation of DivJ (8). However, the biochemical basis of SpmX and DivJ localization to the stalked pole has not been elucidated.

Here, we investigate the mechanism of the ordered recruitment of SpmX and DivJ to the incipient stalked pole. We found that *in vitro*, PopZ directly binds SpmX, that SpmX in turn directly binds DivJ, and that these three proteins are sufficient for polar colocalization when coexpressed heterologously in *Escherichia coli*. SpmX forms higher-order oligomers, suggesting a structural integration between the PopZ and SpmX polymers. Cell cycle-controlled copy number is critical for the geometry of cell pole establishment, as overexpression of SpmX leads to the generation of ectopic growth zones, with the accumulation of SpmX at these sites facilitating the establishment of a PopZ microdomain and the recruitment of the DivJ histidine kinase. The SpmX transmembrane (TM) tether and PopZ are critical for outgrowth of the ectopic cell poles, suggesting that the polar PopZ microdomain coordinates cell polarity through the regulation of cell growth in addition to its established role as founding a signaling protein hub.

## RESULTS

### Reconstitution of the PopZ, SpmX, and DivJ polar complex in *E. coli.*

The polymeric PopZ matrix is positioned together with the PleC phosphatase at the flagellum-bearing pole of the *Caulobacter* swarmer cell (Fig. 1). During the swarmer-to-stalked-cell transition, PleC is released, and SpmX and then the histidine kinase DivJ sequentially colocalize with PopZ at the pole (3, 8). PopZ, SpmX, and DivJ remain at the stalked pole through future generations, while PleC repositions to newly arrived PopZ at the incipient swarmer pole opposite the stalk. Both SpmX and DivJ are delocalized in a *ΔpopZ* background, and DivJ but not PopZ is delocalized in a *ΔspmX* background (6, 8, 9). To determine the minimal requirements for recruitment of DivJ to the stalked pole, we utilized a heterologous *in vivo* system whereby fluorescent fusions of *Caulobacter* proteins were expressed in the *Escherichia coli* strain BL21, which lacks homologs of PopZ, SpmX, and DivJ. This heterologous system has been used successfully to assay protein-protein interactions between PopZ and components of the *Caulobacter* chromosome segregation machinery, ParA and ParB, as well as other pole-localized proteins (7, 12).

**FIG 2:**
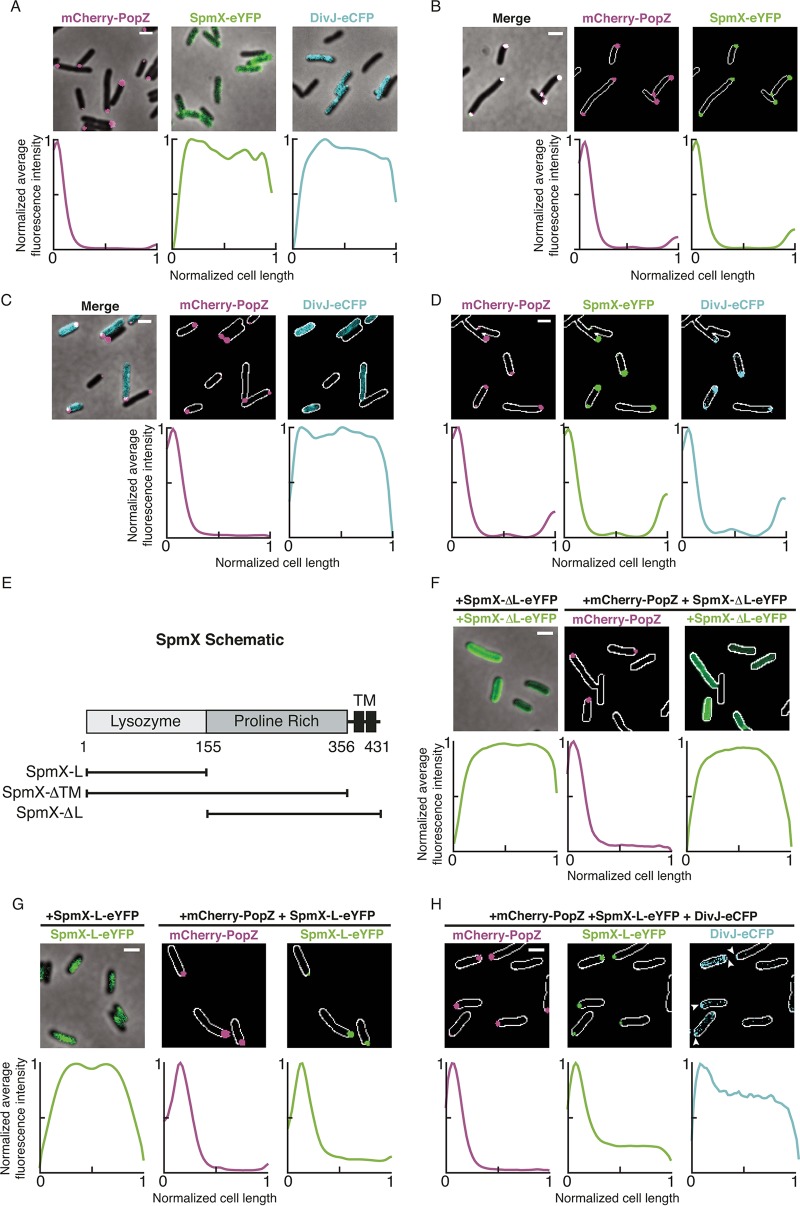
The polar PopZ matrix recruits SpmX, which in turn recruits DivJ, in a heterologous *E. coli* test system for polar protein localization. (A) The *Caulobacter crescentus* fluorescent fusion proteins mCherry-PopZ, SpmX-eYFP, and DivJ-eCFP were assayed for their ability to localize to the cell pole when expressed heterologously in *E. coli*. In each experiment, expression of the fluorescent fusions in *E. coli* was induced for 1 h from a low-copy-number plasmid using either 0.2% l-arabinose (mCherry-PopZ) or 100 μM IPTG (SpmX-eYFP variants and DivJ-eCFP) and imaged via fluorescence microscopy. Plots below each panel quantitatively represent subcellular localization as the normalized fluorescence intensities of each protein over normalized cell lengths. mCherry-PopZ localized to one cell pole, while either SpmX-eYFP or DivJ-eCFP was distributed around the entire cell. (B) The subcellular localizations of mCherry-PopZ and SpmX-eYFP were assayed when coexpressed in *E. coli*. (C and D) The polar localization of DivJ-eCFP was assayed in *E. coli* coexpressing mCherry-PopZ (C) or mCherry-PopZ and SpmX-eYFP (D). (E) Domain schematic showing that WT SpmX contains an N-terminal lysozyme domain, a negatively charged proline-rich intermediate domain, and two C-terminal transmembrane domains. Variants of SpmX that contain just the lysozyme domain (SpmX-L), SpmX missing the transmembrane region (SpmX-ΔTM), and SpmX missing the lysozyme domain (SpmX-ΔL) are indicated. (F and G) The subcellular localizations of the SpmX variants SpmX-ΔL-eYFP (F) and SpmX-L-eYFP (G) expressed in *E. coli* were assayed for their subcellular localization with and without coexpression of mCherry-PopZ. (H) The subcellular localization of DivJ-eCFP was assayed in *E. coli* coexpressing mCherry-PopZ and SpmX-L-eYFP. Arrowheads indicate cell poles with enhanced DivJ signal. Bars, 2 μm.

When *E. coli* bearing a plasmid carrying *mCherry-popZ* under the control of an arabinose promoter was induced with 0.2% l-arabinose for 1 h, mCherry-PopZ localized robustly to one cell pole, as reported previously (Fig. 2A) (5, 6, 12). In contrast, when *E. coli* bearing a plasmid with either *spmX-eyfp* or *divJ-ecfp* was induced with 100 μM isopropyl-β-d-thiogalactopyranoside (IPTG) for 1 h, neither SpmX-enhanced yellow fluorescent protein (eYFP) nor DivJ-enhanced cyan fluorescent protein (eCFP) appeared at the cell pole (Fig. 2A). These data indicate that SpmX and DivJ require additional components not found in *E. coli* for cell pole recruitment.

To determine if PopZ is sufficient to recruit SpmX to the *E. coli* cell pole, we coexpressed mCherry-PopZ and SpmX-eYFP. SpmX-eYFP was found to colocalize with mCherry-PopZ at the cell pole, and a subpopulation of cells established PopZ-SpmX colocalization at both poles (Fig. 2B). Further, a truncated PopZ variant that localizes to the cell poles but does not recruit polar proteins in *Caulobacter* similarly did not recruit SpmX to the *E. coli* cell pole (see Fig. S1A and B in the supplemental material) (7, 10, 12). This finding indicates that the PopZ-SpmX interaction is specific and not due to coaggregation.

10.1128/mBio.02238-16.2 FIG S1  (A) A simplified domain schematic of PopZ illustrates that it contains residues important for specific protein-protein interactions in its N terminus (residues 1 to 102) and contains oligomerization elements in its C terminus (residues 103 to 177). (B) SpmX-eYFP was assayed for polar localization in *E. coli* coexpressing mCherry-PopZ Δ1–102. (C) SpmX-eYFP and DivJ-eCFP coexpressed in *E. coli* without PopZ form colocalized patches, but they do not localize to the cell pole. SpmX-eYFP and DivJ-eCFP were induced with 100 μM IPTG for 1 h. Arrowheads in top panels point to puncta of colocalized eYFP/eCFP signals. Bars, 2 μm. Download FIG S1, EPS file, 0.8 MB. Copyright © 2017 Perez et al. 2017 Perez et al. This content is distributed under the terms of the Creative Commons Attribution 4.0 International license.

In *Caulobacter*, both PopZ and SpmX are necessary for polar localization of DivJ. Consistent with these findings, DivJ did not localize to the *E. coli* cell pole when coexpressed with PopZ alone or with SpmX alone (Fig. 2C; Fig. S1C). However, coexpression of PopZ and SpmX was sufficient to recruit DivJ to the cell poles (Fig. 2D). Together, these data show that PopZ is sufficient to recruit SpmX to the cell pole and that colocalized PopZ-SpmX is sufficient to recruit DivJ.

### The lysozyme-like domain of SpmX is necessary and sufficient for localization to the *E. coli* cell pole.

SpmX contains an N-terminal domain with homology to lysozyme, followed by a proline-rich intermediate domain and two C-terminal TM tethers (Fig. 2E). The putative lysozyme domain of SpmX is required for its localization to the *Caulobacter* cell pole (8). Because Fig. 2B indicates that PopZ recruits SpmX, we asked if the lysozyme-like domain of SpmX contributes to this interaction. We found that when PopZ and SpmX lacking its lysozyme domain were coexpressed in *E. coli*, SpmX-ΔL-eYFP remained diffuse (Fig. 2F), while coexpression of PopZ and SpmX bearing only the lysozyme domain led to the polar localization of SpmX-L-eYFP (Fig. 2G). Thus, the lysozyme-like domain is necessary and sufficient for colocalization with PopZ. SpmX bearing only its lysozyme domain, in the presence of PopZ, partially maintained the ability to recruit DivJ to the cell pole (Fig. 2H). Together, the heterologous expression experiments suggest that the lysozyme-like domain of SpmX is necessary and sufficient to direct it to the cell pole via PopZ but that it is not sufficient to robustly recruit DivJ.

### SpmX directly interacts with both PopZ and DivJ.

**FIG 3:**
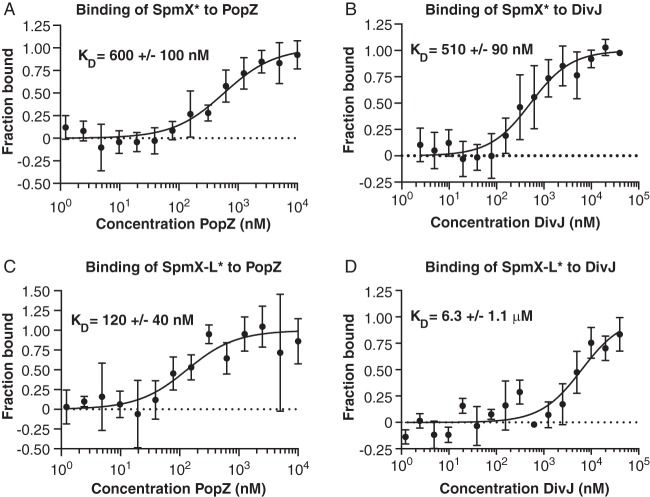
SpmX and SpmX-L directly interact with PopZ and DivJ *in vitro*. (A) The direct binding of purified WT SpmX to PopZ was assessed *in vitro* by microscale thermophoresis. SpmX was fluorescently labeled with Atto-488 dye, indicated by SpmX*. The concentration of SpmX* was held constant at 25 nM while PopZ was titrated in 2-fold serial dilutions against it. The purified proteins were allowed to incubate together at room temperature for 10 min prior to the binding assay. The data report the fraction of SpmX* that is bound at each concentration of PopZ. The binding curve is fitted according to the law of mass action and is described in Text S1 in the supplemental material. (B to D) Direct binding was also assayed between WT SpmX* and DivJ (B), SpmX-L* and PopZ (C), and SpmX-L* and DivJ (D). The maximum concentrations of PopZ and DivJ are 10 μM and 40 μM, respectively. Dissociation constants are listed for each interaction, and each point represents the mean fraction bound of at least 3 independent experiments, with error bars representing the standard deviation.

To demonstrate that SpmX interacts directly with PopZ and DivJ, we purified His-tagged PopZ, SpmX, and DivJ for use in microscale thermophoresis (MST) binding assays (13, 14). SpmX and DivJ were purified without their TM regions. To assay binding of SpmX to PopZ and DivJ, we first sparsely labeled lysine residues on SpmX with Atto-488 dye (indicated as SpmX*). We then measured the PopZ- or DivJ-dependent change in the thermophoresis of SpmX* over a 2-fold serial dilution of either PopZ or DivJ (Fig. 3A and B). Direct binding was observed between SpmX* and PopZ (*K*_*D*_ [equilibrium dissociation constant] = 600 ± 100 nM) and between SpmX* and DivJ (*K*_*D*_ = 510 ± 90 nM).

Dye-labeled SpmX-L*, bearing only the lysozyme domain, also directly bound to PopZ and DivJ but with affinities altered from those observed with wild-type (WT) SpmX* (Fig. 3C and D). The 10-fold-weakened affinity of SpmX-L* for DivJ (*K*_*D*_ = 6.3 ± 1.1 μM) is consistent with the reduced capacity of SpmX-L-eYFP to recruit DivJ when coexpressed in *E. coli* (Fig. 2H). The affinity of SpmX-L for PopZ was 5-fold stronger (*K*_*D*_ = 120 ± 40 nM) than that of WT. The proline-rich domain of SpmX is highly negatively charged, as is PopZ, suggesting that the interaction between SpmX-L and PopZ may be strengthened when this electrostatic repulsion is alleviated.

Surface plasmon resonance (SPR) experiments corroborated our finding that PopZ directly binds to both WT SpmX and SpmX-L (Fig. S2). PopZ was immobilized onto a biosensor chip, and 2-fold serial dilutions of WT SpmX or SpmX-L were injected over PopZ to assay for binding. The overall response of SpmX-L binding to PopZ was approximately 2-fold higher than that of WT SpmX at comparable concentrations, consistent with the tighter *K*_*D*_ of SpmX-L binding to PopZ as measured via MST.

10.1128/mBio.02238-16.3 FIG S2  Purified WT SpmX (residues 1 to 355) and SpmX-L (residues 1 to 155) directly bind to PopZ, as measured by surface plasmon resonance (A and B, respectively). Purified PopZ was immobilized on the sensor chip, and SpmX variants were injected over PopZ at increasing concentrations, with washes to remove bound SpmX between injections. Download FIG S2, EPS file, 0.6 MB. Copyright © 2017 Perez et al. 2017 Perez et al. This content is distributed under the terms of the Creative Commons Attribution 4.0 International license.

Cumulatively, our results demonstrate a hierarchical localization pathway whereby PopZ at the flagellum-bearing cell pole serves to recruit SpmX via a direct interaction during the swarmer-to-stalked-cell transition. SpmX bound to PopZ then recruits DivJ to the cell pole via a second interaction. Thus, SpmX serves as a bridge between the polar PopZ matrix and the DivJ kinase in order to localize DivJ to a specific cell pole.

### Function of SpmX domains in *Caulobacter.*

To interrogate the functions of each domain of SpmX in *Caulobacter*, we examined the phenotypes of *Caulobacter* cells expressing SpmX domain deletion alleles fused to eYFP, from the native *spmX* promoter, and as the only copy of SpmX present in the cell. We assayed the subcellular localization of WT and two mutant SpmX alleles fused to eYFP: SpmX-L and SpmX missing its TM domains (SpmX-ΔTM) (Fig. S3A). Wild-type SpmX-eYFP displayed the previously reported nascent stalked pole localization pattern (8). In addition to the stalked pole signal, we also observed 18% of cells displaying a second, less intense focus at the new cell pole (Fig. S3A).

10.1128/mBio.02238-16.4 FIG S3  (A) The subcellular localizations of WT SpmX-eYFP, SpmX-L-eYFP, and SpmX-ΔTM-eYFP were imaged in *C. crescentus*. Each SpmX variant was expressed from the native *spmX* locus as the only copy of SpmX. The white arrowhead indicates a minicell, and the black arrowhead indicates a cell displaying a second SpmX focus at the new cell pole. Localization was quantified as normalized fluorescence intensities, plotted along a normalized cell length axis, with 0 indicating the stalked pole. SpmX-L-eYFP exhibited both polar localization and a diffuse pattern of subcellular positioning, while SpmX-ΔTM-eYFP exhibited bipolar localization. (B) The subcellular localization of DivJ-mCherry was assayed in the *C. crescentus* strains from the previous panel, expressing SpmX-eYFP variants. DivJ-mCherry was expressed from its native promoter as the sole copy of DivJ. DivJ-mCherry colocalized with WT SpmX-eYFP at the cell pole but did not colocalize with the SpmX variant SpmX-L-eYFP or SpmX-ΔTM-eYFP. All bars, 2 μm. (C) Model for how deletion of the SpmX transmembrane tether affects the localization of SpmX and DivJ in *C. crescentus*. When the membrane tether is present, SpmX and DivJ can interact on the membrane while also contacting the PopZ matrix. Without the membrane tether, SpmX can interact with PopZ molecules that are further from the cell membrane, represented by the cartoon SpmX accumulations moving further from the membrane while staying within the PopZ matrix. This repositioning of SpmX within the PopZ matrix may decrease its effectiveness for recruiting DivJ to the stalked pole. Without its interaction with DivJ, SpmX variants lacking its transmembrane domain may now be free to localize to both PopZ foci. (D) Domain schematic showing that WT SpmX contains an N-terminal lysozyme domain, a negatively charged proline-rich intermediate domain, and two C-terminal transmembrane domains. Variants of SpmX that contain just the lysozyme domain (SpmX-L), SpmX missing the transmembrane region (SpmX-ΔTM), and SpmX missing the lysozyme domain (SpmX-ΔL) are indicated. Download FIG S3, EPS file, 1.2 MB. Copyright © 2017 Perez et al. 2017 Perez et al. This content is distributed under the terms of the Creative Commons Attribution 4.0 International license.

Replacing native SpmX with the truncated variant SpmX-L-eYFP or SpmX-ΔTM-eYFP yielded morphological defects, including filamentation and the formation of minicells (Fig. S3A). Additionally, SpmX-L-eYFP and SpmX-ΔTM-eYFP formed bipolar foci in 54% and 59% of cells, respectively, indicating that the SpmX TM domains contribute to unipolar SpmX localization. Western blotting assays showed that the relative levels of the SpmX-L-eYFP- and SpmX-ΔTM-eYFP-tagged mutant variants were slightly higher than WT (Fig. S4A and B). Despite robust recruitment to PopZ, these SpmX variants failed to recruit DivJ-mCherry to the *Caulobacter* cell pole (Fig. S3B and C), consistent with previous findings (8). Even though the *in vitro* binding assays showed that purified SpmX does not require its TM domains to interact with DivJ *in vitro* (Fig. 3B and D), the requirement for the TM domains *in vivo* is likely due to the space-filling nature of the PopZ polar microdomain (7, 15) (Text S1). These results demonstrate that the SpmX lysozyme domain alone cannot complement WT SpmX function, despite its ability to directly bind to both PopZ and DivJ.

10.1128/mBio.02238-16.5 FIG S4  (A and B) Western blot assays of *Caulobacter* strains expressing the indicated SpmX variants as the sole copy of *spmX*. Whole-cell lysates were separated via SDS-PAGE, transferred to a nitrocellulose membrane, and probed with anti-SpmX (A) or anti-GFP (B) antisera. The SpmX antibody was raised against a peptide in the proline-rich domain and does not detect the lysozyme domain. Cells harboring the SpmX variant E19R were slower growing than *ΔspmX* cells but were not stably maintained in *Caulobacter* at native expression levels. Note that the SpmX-eYFP fusion has a predicted molecular mass of 74 kDa but migrates at an apparent molecular mass of approximately 95 kDa, likely due to the proline-rich domain. The blots displayed are representative of three independent experiments. (C) A Western blot shows comparable protein levels for the xylose-induced overexpression of SpmX-eYFP and that of SpmX E19R. The genotypes of the cells used for each lane are shown directly below the Western blot. Additional details about the antibodies used are provided in Text S1. (D) Coomassie blue-stained SDS-PAGE fractions collected from size exclusion chromatography analysis of purified SpmX (Fig. 6A) confirm that impurities did not yield confounding peaks in the size exclusion analysis. Purified SpmX migrates at least 10 kDa above its predicted molecular mass in denaturing gel electrophoresis experiments, also observed in Western blots (B). Download FIG S4, TIF file, 1.3 MB. Copyright © 2017 Perez et al. 2017 Perez et al. This content is distributed under the terms of the Creative Commons Attribution 4.0 International license.

10.1128/mBio.02238-16.1 TEXT S1  Supplemental discussion, methods, and two tables. Download TEXT S1, DOCX file, 0.1 MB. Copyright © 2017 Perez et al. 2017 Perez et al. This content is distributed under the terms of the Creative Commons Attribution 4.0 International license.

### Point mutations in the lysozyme-like domain of SpmX affect SpmX polar localization and function in *Caulobacter.*

Lysozyme catalytic activity requires a conserved glutamic acid residue in the active site (16–18). The SpmX lysozyme-like domain conserves this residue at position E19 (Fig. S5A). Despite extensive efforts, we have not been able to detect SpmX lysozyme activity *in vitro*, though we were able to observe lysozyme activity for hen egg white lysozyme in identical assays (Fig. S5C). Nevertheless, we asked if mutation of E19 would affect SpmX localization and function *in vivo*. We mutated E19 to either alanine or arginine and expressed the mutant alleles as eYFP fusions as the sole chromosomal copy of *spmX* at the native locus (Fig. S5B). SpmX E19A-eYFP displayed bipolar localization in 59% of cells (compared to 18% for WT SpmX-eYFP) and mild cell filamentation defects (Fig. S5B). Cells harboring replacements of *spmX* with the point mutant SpmX E19R-eYFP did not maintain the protein at detectable levels (Fig. S4B), suggesting that it is degraded. Purified SpmX E19R failed to interact with either PopZ or DivJ *in vitro* via MST binding assays (Fig. S6A to C), and a homology model for the SpmX lysozyme domain, based on T4 endolysin, predicts that E19 participates in an electrostatic interaction that may contribute to the protein’s stability and interactions (Fig. S6E). These data suggest that the catalytic region of this lysozyme domain is critical for normal interaction with PopZ and DivJ.

10.1128/mBio.02238-16.6 FIG S5  (A) The peptide sequence of the putative lysozyme-like domain of SpmX is aligned with hen egg white lysozyme (HEWL) and phage T4 endolysin. Residues are highlighted according to charge and hydrophobicity (purple, positive charge; blue, negative charge; red, hydrophobic; green, neutral). Stars indicate conserved residues of HEWL and T4 endolysin, and an arrow indicates the conserved glutamate residue critical to catalysis in HEWL and T4 endolysin. (B) The subcellular localization of SpmX-E19A-eYFP was assayed in *C. crescentus* expressing the indicated point mutant from the native promoter as the only copy of *spmX*. Arrowheads in the E19A images point to mislocalized SpmX-eYFP variant foci. Native expression levels of the E19R protein were not sufficient to maintain the protein (also see Fig. S3). Bar, 2 μm. (C) Lyophilized *Micrococcus lysodeikticus* cells were resuspended in buffer and treated with buffer (black line), 20 μM SpmX (blue line), or 20 μM hen egg white lysozyme (HEWL, red line). The turbidity of solution was monitored by absorbance at 450 nm once a minute over 30 min to monitor degradation of peptidoglycan. Each point represents the average from two experiments, with error bars displaying the range of the data. Download FIG S5, EPS file, 1.1 MB. Copyright © 2017 Perez et al. 2017 Perez et al. This content is distributed under the terms of the Creative Commons Attribution 4.0 International license.

10.1128/mBio.02238-16.7 FIG S6  (A to C) The direct binding of purified Atto-488 dye-labeled SpmX (E19R) to PopZ, WT SpmX, and DivJ was assessed *in vitro* by microscale thermophoresis as described in the Fig. 3 legend and Text S1. The concentration of SpmX (E19R)* was held constant at 50 nM, while PopZ was titrated in 2-fold serial dilutions against it. Because these data could not be meaningfully fitted to binding curves, and hence reported as fraction bound as in Fig. 3, the data are reported as normalized fluorescence. This output represents the change in steady-state fluorescence of SpmX (E19R)* induced by the heating period of the MST experiment as a function of the concentration of the tested ligand. (D) We display an equivalently processed data set for WT SpmX, which does directly bind to PopZ. Each data point represents the mean from at least 3 independent experiments. Error bars are not displayed, as day-to-day differences in relative mobility induced by heating are sometimes large compared to the mobility change induced by the ligand within a single experiment. (E) A homology model of the lysozyme domain of SpmX, based on T4 endolysin. The model highlights the predicted interaction between residues E19 and R140, suggesting that disruption of this interaction contributes to the phenotypes associated with E19. Download FIG S6, EPS file, 1.6 MB. Copyright © 2017 Perez et al. 2017 Perez et al. This content is distributed under the terms of the Creative Commons Attribution 4.0 International license.

### SpmX constitutive overexpression generates ectopic growth zones.

**FIG 4:**
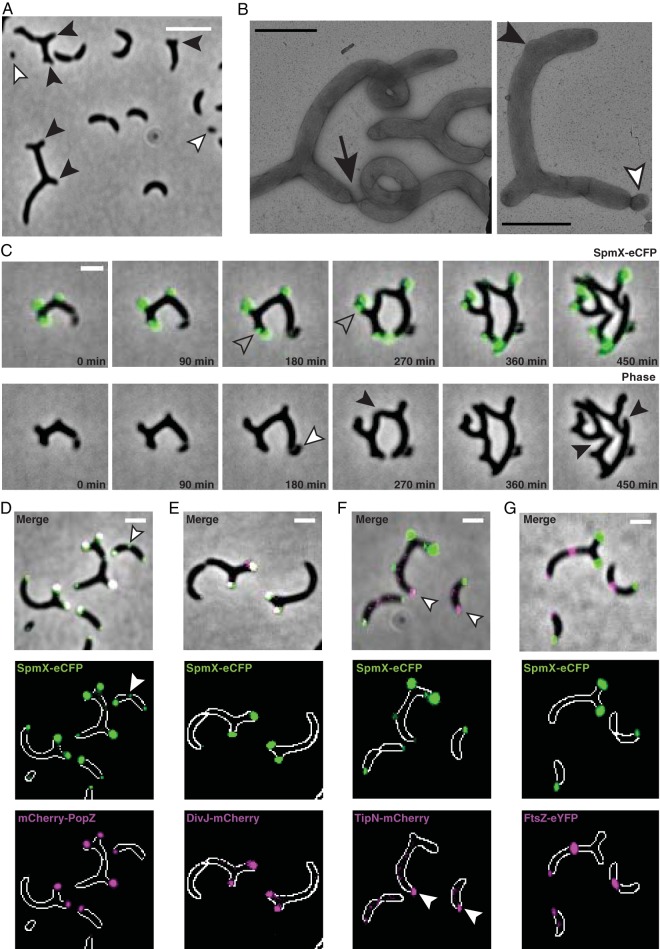
Constitutive overexpression of SpmX causes formation of ectopic cell poles. (A) Overexpression of SpmX from the *xylX* promoter causes a subpopulation of cells to grow ectopic cell poles, shown in a phase-contrast micrograph. Black arrowheads point to cells displaying ectopic pole growth. White arrowheads point to minicells. Bar, 5 μm. (B) Cells overexpressing SpmX were visualized by transmission electron microscopy of negatively stained samples. The black arrowhead indicates the initiation of a lateral growth zone, and the white arrowhead indicates budding of a minicell. The arrow points to a constriction event. (C) Time-lapse imaging of cells overexpressing SpmX-eCFP from the chromosomal *xylX* promoter shows that cells with ectopic poles are viable and capable of cell division. Cells were spotted onto agarose pads and imaged at the indicated time intervals. Open arrowheads point to ectopic SpmX-eCFP foci that precede the formation of ectopic poles. Black arrowheads point to division events. The white arrowhead points to a budded minicell. (D to G) The subcellular localizations of mCherry-PopZ, DivJ-mCherry, TipN-mCherry, and FtsZ-eYFP were observed during SpmX-eCFP overexpression. Arrowheads indicate lateral accumulation of SpmX-eCFP (D) or accumulations of TipN at cell poles without SpmX-eCFP present (F). Bars, 2 μm (B to G). Expression of SpmX from the *xylX* locus was induced for 18 h using 0.3% xylose in all panels. FtsZ-eYFP was expressed from the *vanA* locus for 30 min with 50 μm vanillate prior to imaging.

The majority of SpmX remains at the stalked pole for the duration of the cell cycle (Fig. 1A) (8, 11). We reasoned that constitutive overexpression of SpmX could disrupt its asymmetric polar localization and normal function. Indeed, inducing *spmX* expression from the chromosomal *xylX* promoter was sufficient to produce minicells and, more strikingly, to initiate ectopic growth zones in ~10% of cells (Fig. 4A). Transmission electron microscopy of these cells indicated that these ectopic growth zones had diameters and shapes resembling those of wild-type cells (Fig. 4B).

To determine if overexpressed SpmX localized to the ectopic poles, we performed time-lapse microscopy of cells overexpressing *spmX-ecfp* (Fig. 4C). SpmX-eCFP accumulated at the cell poles in cells displaying ectopic growth. Many of these cells had one cell pole with strikingly diminished SpmX-eCFP intensity, including a subset of poles lacking SpmX that budded off as minicells. Critically, we observed the ectopic accumulation of SpmX-eCFP signal in nonpolar regions of the cell that preceded the formation of ectopic pole growth (Fig. 4C, open arrowheads), and cells extending from ectopic growth zones were capable of division for multiple replication cycles (Fig. 4C, black arrowheads). Subsequent progeny continued to produce ectopic growth zones, indicating that they are capable of sustaining growth while exhibiting markedly altered axes of symmetry.

### Polar identity of ectopic poles is maintained during SpmX overexpression.

We visualized cells coexpressing SpmX-eCFP with other fluorescently labeled proteins normally positioned at the cell poles. We observed mCherry-PopZ colocalization with overexpressed SpmX-eCFP at many cell poles (Fig. 4D). Specifically, cells containing SpmX-eCFP accumulations at lateral regions sometimes colocalized with PopZ but PopZ never arrived at lateral regions without SpmX (Fig. S7). This finding suggests that during SpmX overexpression, SpmX can recruit PopZ to lateral sites prior to growth of an ectopic pole.

10.1128/mBio.02238-16.8 FIG S7  (A) A panel of *Caulobacter* cells overexpressing SpmX-eCFP shows that many cells maintain a single pole with significantly diminished SpmX-eCFP signal (black arrowheads), indicating that the new pole maintains its protein composition even during SpmX overexpression. (B) SpmX-eCFP accumulation outside the cell pole occurs both with and without PopZ colocalization at those sites (white arrowheads indicate lateral SpmX/PopZ foci), indicating that SpmX-eCFP can recruit PopZ to nonpolar sites when overexpressed. Bars, 2 μm. Download FIG S7, EPS file, 0.5 MB. Copyright © 2017 Perez et al. 2017 Perez et al. This content is distributed under the terms of the Creative Commons Attribution 4.0 International license.

To determine the identities of the ectopic poles, we examined the subcellular localization of the mCherry-tagged stalked and swarmer pole markers DivJ and TipN, respectively. DivJ-mCherry foci colocalized with SpmX-eCFP at the ectopic cell poles (Fig. 4E), while TipN-mCherry did not (Fig. 4F, white arrowheads). Strikingly, cell poles that lacked SpmX-eCFP signal did not contain a corresponding DivJ-mCherry focus but did contain a polar focus of TipN-mCherry (Fig. 4E and F), and we did not observe multiple polar TipN-mCherry accumulations. These data indicate that, during SpmX overexpression, most poles contain a stalked pole protein composition but suggest that one “swarmer pole” composition is maintained.

We additionally found that the division plane marker FtsZ-eYFP localized as a discrete band at medial positions of the cell body (Fig. 4G), with minor accumulations at the new pole, as has been previously observed (19). Cumulatively, our data demonstrate that cells undergoing abnormal growth during SpmX overexpression retain polar identity and the ability to divide and are able to sustain growth.

### PopZ is critical for ectopic pole growth during SpmX overexpression.

**FIG 5:**
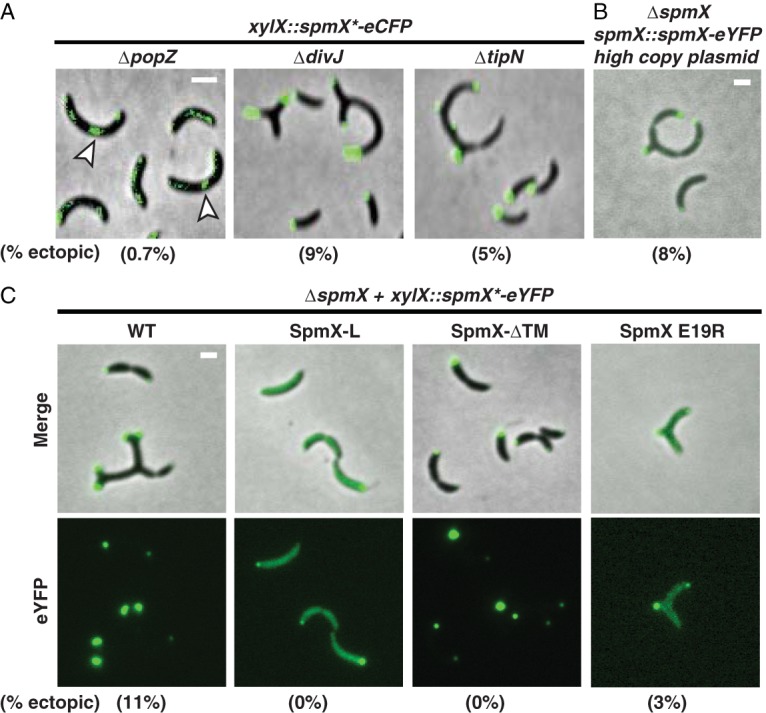
Generation of ectopic poles requires PopZ and the SpmX transmembrane domains. (A) Cell morphology and SpmX-eCFP subcellular localization were assayed during SpmX-eCFP overexpression from the *xylX* locus in *ΔpopZ*, *ΔdivJ*, and *ΔtipN* strain backgrounds. White arrowheads point to lateral SpmX-eCFP foci. (B) SpmX-eYFP was expressed from its native promoter on a high-copy-number plasmid in a *ΔspmX* background. (C) The indicated mutant variants of SpmX fused to eYFP were overexpressed from the *xylX* locus in a *ΔspmX* background (left panel). For panels A and C, expression of SpmX from the *xylX* locus was induced for 18 h using 0.3% xylose. For all panels, the percentage of cells displaying ectopic poles is given in parentheses. Bars, 2 μm.

Given that SpmX interacts with both PopZ and DivJ, we asked whether formation of ectopic poles driven by SpmX overexpression requires PopZ and/or DivJ. Accordingly, we overexpressed SpmX-eCFP in either a *ΔpopZ* or a *ΔdivJ* background. In the absence of DivJ, SpmX-eCFP overexpression still induced ectopic pole formation (9% of cells, compared to ~10% in an otherwise isogenic background) (Fig. 5A), indicating that DivJ is not required for ectopic growth under these conditions. However, ectopic poles were very rarely observed when SpmX-eCFP was overexpressed in the absence of PopZ (0.7% of cells). The SpmX-eCFP signal under this condition exhibited a patchy distribution throughout the cell, with large clusters of signal present at variable subcellular locations (Fig. 5A). Thus, PopZ is critical for ectopic pole formation during SpmX overexpression and is needed to cluster SpmX into tight foci as visualized by fluorescence microscopy. We propose that SpmX overexpression facilitates the formation of an ectopically placed SpmX/PopZ complex. This complex then redirects cell growth to initiate the formation of a new growth zone.

It has been reported that overexpression of TipN also results in the growth of ectopic poles (20). We asked if SpmX overexpression induces ectopic pole growth via a mechanism that is distinct from that caused by TipN overexpression. Accordingly, we overexpressed SpmX-eCFP in a *ΔtipN* background and observed that ectopic pole growth still occurred in the absence of TipN (5% of cells [Fig. 5A, right]). While there are indeed fewer cells displaying ectopic poles in a *ΔtipN* background (5% compared to ~10% for WT), the observation that TipN-mCherry specifically does not colocalize at ectopic poles with SpmX-eCFP (Fig. 4F) suggests that TipN does not play a direct or critical role in the formation of SpmX-seeded ectopic growth zones.

Because the production of the SpmX protein is tightly cell cycle regulated during normal *Caulobacter* growth (8, 11, 21), we asked if the constitutive timing of expression contributed to the formation of ectopic cell poles. Overexpressing *spmX-eYFP* from its native promoter on a high-copy-number plasmid in a *ΔspmX* background generated ectopic poles in 8% of cells, compared to approximately 10% when *spmX-eYFP* was expressed from the chromosomal *xylX* locus (Fig. 5B). Given this relatively small difference, these data indicate that the expression levels of overall levels of SpmX and not its timing of expression are critical for maintaining an axis of symmetry.

### The transmembrane tethers of SpmX are necessary to generate ectopic poles.

To interrogate the molecular features of SpmX needed for the formation of ectopic growth zones, we overexpressed three SpmX alleles (SpmX E19R, SpmX-L, and SpmX-ΔTM) fused to eYFP via the chromosomal xylose promoter in a *ΔspmX* background. Overexpression of both SpmX-ΔTM-eYFP and SpmX-L-eYFP failed to produce cells undergoing ectopic growth (0% of cells for both alleles) (Fig. 5C), indicating that the SpmX transmembrane domains are necessary for ectopic pole growth. We also assayed the effect of the mutation E19R on the ability of overexpressed SpmX to form ectopic growth zones. While the SpmX E19R variant was not stable at native expression levels, overexpression stabilized the variant as weak polar accumulations (Fig. S4B and C; Fig. 5C), possibly due to protection from defects in oligomerization, as described below. Overexpression of SpmX E19R-eYFP yielded cells with ectopic growth zones but at lower levels than with WT (3% to 11%, respectively) (Fig. 5C), perhaps reflecting the impaired ability of this SpmX mutant to interact with PopZ *in vitro*. The drop in ectopic pole formation and reduced capacity to localize to the cell poles suggests that SpmX E19R has a diminished ability to generate ectopic poles upon overexpression, perhaps due to its impaired ability to interact with PopZ (Fig. S6).

### SpmX oligomerizes via interactions in the lysozyme and proline-rich domains.

**FIG 6:**
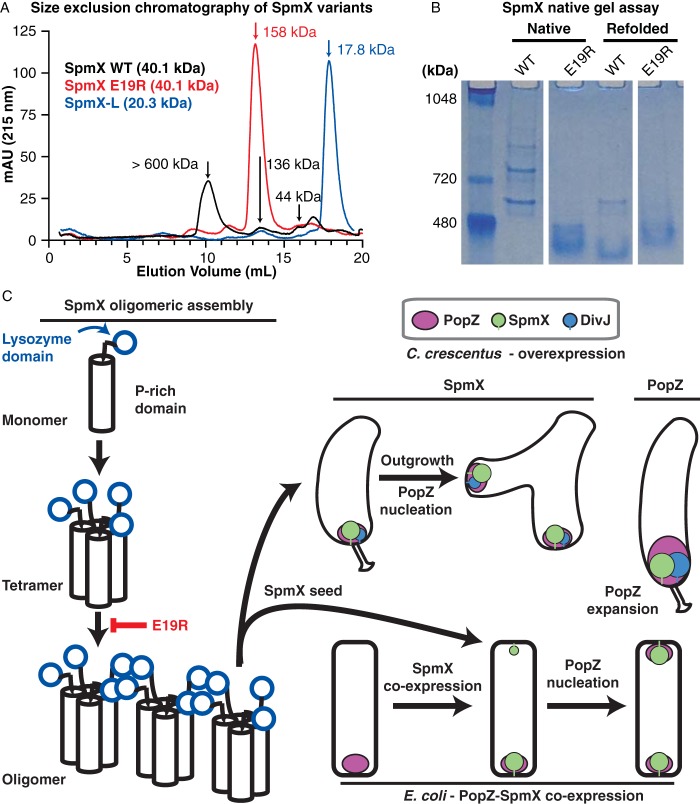
SpmX oligomerizes *in vitro* through its lysozyme-like domain. (A) Analytical size exclusion chromatography was used to measure the apparent molecular mass of purified WT SpmX (black line) and the variants SpmX E19R (red line) and SpmX-L (blue line). Absorbance at 215 nm versus elution volume is plotted. The indicated molecular masses of each peak were determined by comparison to the elution volume of protein standards. A representative trace is shown from two independent replicates of each size separation. (B) The folding and oligomerization of purified WT SpmX and SpmX-E19R were assessed *in vitro* via native gel analysis. The refolded samples were first diluted 5-fold using 8 M urea and incubated at room temperature for 1 h before being refolded in buffer for 2 h at 4°C; for the native samples, the proteins were diluted similarly in buffer. The proteins were then subjected to nondenaturing gel electrophoresis at 4°C and subsequently stained with Coomassie blue. A molecular mass standard (in kilodaltons) is shown in the leftmost lane. (C) Model of SpmX oligomerization and subcellular localization. The proline-rich domain of SpmX mediates assembly of SpmX into tetramers. These tetramers further assemble into higher-ordered structures via contacts through the lysozyme-like domain. The SpmX mutation E19R blocks this step in the SpmX assembly pathway. Overexpression of SpmX in *Caulobacter* facilitates ectopic localization of SpmX to lateral regions of the cell, where it recruits PopZ and leads to the growth of ectopic cell poles. Similarly, coexpression of SpmX and PopZ in *E. coli* leads to nucleation of SpmX at the cell pole lacking PopZ, where it serves to recruit PopZ, which is otherwise monopolar. Overexpression of PopZ in *Caulobacter* causes expansion of the PopZ matrix but does not nucleate ectopic accumulations.

The observation that the SpmX-eCFP overexpression resulted in accumulations of lateral SpmX foci in the absence of PopZ (Fig. 5A) suggested that SpmX might self-associate, providing a mechanism to seed ectopic growth zones. To test this, we measured the apparent molecular mass of purified SpmX variants by analytical size exclusion chromatography. While the predicted monomeric molecular mass of WT SpmX is 40.1 kDa, the majority of WT SpmX eluted in the void volume, suggestive of a molecular mass greater than 600 kDa, with smaller peaks corresponding to estimated molecular masses of 136 kDa and 44 kDa (Fig. 6A). Strikingly, the SpmX E19R variant eluted primarily at an apparent molecular mass of 158 kDa, close to the predicted molecular mass of a tetrameric SpmX species. SpmX-L eluted as a monomer, indicating that the proline-rich domain is necessary for oligomeric assembly (Fig. 6A). While secondary structure predictions indicate that much of this region forms a random coil, a 19-residue stretch in this region is free of prolines and predicted to contain an extended alpha-helix that could assist in oligomerization (Fig. S8) (22, 23).

10.1128/mBio.02238-16.9 FIG S8  (A) A PSIPRED secondary structure prediction for SpmX maps out predicted regions of secondary structure and disorder. The figure highlights three types of secondary structure categorization: alpha-helices (pink column), beta-strands (yellow arrow), or random coil (black line). Higher blue bars indicate a higher confidence prediction. The predicted secondary structure element (Helix, Strand, Coil) is also abbreviated alphabetically over the peptide sequence of SpmX. The predicted secondary structure in the first 150 residues is similar to known lysozyme structures, two transmembrane helices are identified near the C terminus, and a locally structured region from residues 215 to 228 may participate in oligomerization. (B) A DISOPRED visual representation of predicted secondary structure disorder highlights structured elements in the lysozyme domain, the predicted alpha-helical stretch in the proline-rich domain, and the transmembrane helices at the C terminus. Both the PSIPRED and DISOPRED servers are available at http://bioinf.cs.ucl.ac.uk/psipred/. Download FIG S8, EPS file, 1.5 MB. Copyright © 2017 Perez et al. 2017 Perez et al. This content is distributed under the terms of the Creative Commons Attribution 4.0 International license.

Native gel analysis of the same proteins showed that WT SpmX migrated in a ladder-like pattern composed of discrete pairs of bands (Fig. 6B). While the elution of SpmX complexes in the void volume of the gel filtration column (Fig. 6A) could suggest that this protein exists as nonfunctional aggregates, the well-resolved, repeated addition pattern of the native gel indicates that the protein exists in discrete species formed by regularly sized additions. And, though the displayed molecular mass standards of the native gel do not evenly account for charge or shape of distinct substrates under nondenaturing conditions, these species were centered near the 720-kDa marker. SpmX E19R ran as a single smeared band at a lower apparent molecular mass than the WT, consistent with a defect in higher-order assembly. Purified PopZ protein is capable of refolding into its native oligomeric structure after denaturation (5, 6). We asked whether SpmX also has this property. Following denaturation and refolding in nondenaturing buffer, native gel electrophoresis showed that WT SpmX partially reformed the fastest-migrating native band, while a majority of the protein migrated as a band similar to nondenatured SpmX E19R. Refolded SpmX E19R migrated in a pattern similar to its nondenatured form. These results indicate that the mutation E19R in the lysozyme-like domain of SpmX specifically disrupts the assembly of higher-order oligomeric structures and suggest that the intermediate assembly product trapped by E19R is capable of refolding after denaturation.

To further validate the SpmX self-association, we assayed the binding of purified, dye-labeled WT SpmX* to unlabeled SpmX by MST. We observed a single oligomeric transition of labeled SpmX* binding to itself and a weakened interaction between SpmX-L* and SpmX (Fig. S9A and B) (*K*_*D*_ = 360 ± 50 nM and 630 ± 70 nM, respectively). SpmX E19R did not show any binding to WT SpmX (Fig. S6). These measured interactions between the lysozyme-like domain and the WT SpmX species indicates that the lysozyme-like domain of SpmX makes contact with a WT SpmX species, suggesting that it can incorporate into an oligomeric structure when the full-length protein is present and that compromising this domain via mutagenesis partially disrupts SpmX self-association. The finding that SpmX undergoes one observable binding transition in MST experiments suggests that the assembly into an intermediate state occurs at a concentration below the 25 nM level at which we probed oligomerization with SpmX*, consistent with our inability to observe an E19R oligomeric transition. Notably, the SpmX E19R mutant displays a reduced ability to form ectopic foci and cell poles when overexpressed *in vivo* (Fig. 5C), suggesting that its ability to form multimers is compromised and that this oligomerization contributes to SpmX function.

10.1128/mBio.02238-16.10 FIG S9  (A) The direct binding of purified fluorescently labeled WT SpmX (SpmX*) to itself was assessed *in vitro* by microscale thermophoresis. Binding assays were conducted and are reported as described in the legend to Fig. 3, but with the adjustment that unlabeled SpmX was titrated against labeled SpmX*. (B) Direct binding of fluorescently labeled SpmX-L (SpmX-L*) to WT SpmX was measured by microscale thermophoresis. Download FIG S9, EPS file, 0.6 MB. Copyright © 2017 Perez et al. 2017 Perez et al. This content is distributed under the terms of the Creative Commons Attribution 4.0 International license.

Cumulatively, the *in vitro* self-association experiments suggest that SpmX forms oligomers that assemble through a multistep process (Fig. 6C). The proline-rich domain is strictly required for oligomerization, and the mutation E19R in the lysozyme-like domain disrupts further higher-order assembly, suggesting that this domain mediates additional contacts. Further, comparison of the SpmX variants’ ability to form oligomers *in vitro* with their relative ability to form fluorescent foci *in vivo* suggests a possible mechanism for both the polar localization of SpmX and seeding of new SpmX-PopZ accumulations and the initiation of ectopic cell pole growth when SpmX is overexpressed.

## DISCUSSION

Here, we have shown that the lysozyme-like domain of SpmX mediates the ordered assembly of a signaling complex through direct interactions with the PopZ polymeric matrix and the DivJ histidine kinase. In addition to mediating contact with PopZ and DivJ, the lysozyme-like domain contributes to oligomerization of SpmX. When overexpressed, SpmX seeds ectopic cell poles, recruiting the DivJ histidine kinase, with outgrowth of these poles dependent on the establishment of a localized PopZ microdomain. The PopZ-SpmX complex thus coordinates cellular polarity through its coordinated regulation of cell fate and localized cell growth.

### The repurposed lysozyme-like factor SpmX bridges the assembly of a microdomain signaling complex with the PopZ polymeric network.

SpmX and DivJ are produced sequentially at the beginning of the swarmer-to-stalked-cell transition (8). We recapitulated the polar localization dependencies of these proteins in an *in vivo* heterologous *E. coli* expression system (Fig. 2D), indicating that SpmX forms a bridge between PopZ and DivJ in the polar complex. Further, we demonstrated that purified SpmX directly interacts with both PopZ and DivJ *in vitro* (Fig. 3). We conclude that SpmX directly bridges PopZ to DivJ in *Caulobacter*, localizing the kinase at the incipient stalked pole.

The N-terminal lysozyme-like domain of SpmX has been shown to be a polar localization determinant for SpmX in *Caulobacter* (8). Consistent with this result, we show that the SpmX lysozyme-like domain is sufficient for colocalization with PopZ at the heterologous *E. coli* cell pole *in vivo* as well as for a direct interaction with PopZ *in vitro* (Fig. 2G and 3C). We have not detected *in vitro* catalytic activity for purified SpmX constructs, but we did find that mutation of the conserved, catalytic residue E19 disrupted the abilities of SpmX to localize to one pole or to form ectopic poles upon overexpression *in vivo* (Fig. 5C; also see Fig. S5 in the supplemental material). While we found that WT SpmX forms oligomers *in vitro*, the mutant SpmX E19R cannot oligomerize into the highest-order structures observed in size exclusion chromatography and native gel electrophoresis (Fig. 6A and B). This mutant is also incapable of interacting with both PopZ and DivJ, raising the possibility that SpmX oligomerization is important for its interaction with other proteins. Together, these data suggest that the lysozyme-like fold of SpmX has been repurposed as a mediator of oligomeric assembly and protein recruitment. We additionally found that the proline-rich domain of SpmX was necessary for initial oligomerization into a tetrameric state, as indicated by the comparison of the apparent molecular weights (MWs) of the full-length E19R point mutant and the isolated lysozyme-like domain (Fig. 6A and C). This initial oligomerization event may occur through an isolated, extended α-helix, which is predicted to be an island of local structure amid the otherwise disordered region (Fig. S8). Holmes et al. recently suggested that the disordered proline-rich region of PopZ critically contributes to its ability to localize up to 11 different protein substrates *in vivo* (7), and conservation of this motif in SpmX further supports a critical role for it in organizing polarity factors.

### Models for the generation of ectopic growth zones during SpmX overexpression.

Two primary models can explain the mechanism by which overproduction of SpmX generates ectopic poles: SpmX is the central actor in the generation of an ectopic pole or lateral aggregates of SpmX recruit additional factors that build the new poles. The lysozyme-like domain suggests that SpmX could degrade or perhaps modify peptidoglycan (PG) through a process such as transglycosylation (17), directly generating outgrowths by acting on the cell wall. While it has been suggested that SpmX exists in the periplasm (8), where lysozyme typically acts, SpmX lacks periplasm secretion motifs via either the Sec or twin-arginine transport pathways (24, 25). Further, the direct interactions between SpmX-L and the cytoplasmic protein PopZ and the cytoplasmic portion of DivJ (Fig. 3C and D) suggest that SpmX likely exists largely in the cytoplasm, though it remains possible that there is an additional method for transiently transporting SpmX to the periplasm. Alternatively, SpmX may participate in an upstream step in PG assembly that occurs in the cytoplasm prior to lipid II flipping (26). The fact that the E19R mutant still retains some ability to generate ectopic poles strongly argues against this hypothesis. However, because we have no evidence that the SpmX lysozyme domain has catalytic activity, we favor the hypothesis that aggregates of SpmX attract the formation of a PopZ microdomain and recruit factors involved in cell wall growth to create new regions of localized outgrowth.

Cell growth normally occurs via the addition of new PG at two cellular locations in *Caulobacter*: at the midcell and at the base of the stalk, where SpmX typically resides (27–29). Specifically, SpmX may coopt cell growth machinery normally dedicated to stalk biogenesis and repurpose it to build new cell poles. Further, overexpression of SpmX may inactivate the transcription factor TacA, which promotes stalk biogenesis (30), instead forcing this normally localized cell wall growth to ectopic sites. Depletion of the cytoskeletal protein MreB or the PG synthase RodA (31) has been shown to yield ectopic poles in *Caulobacter* at the expense of normal stalk biogenesis (32).

While SpmX is important for stalk formation and placement in the related genus *Asticcacaulis* (33), it is not in *Caulobacter* (8). However, PopZ is a critical regulator of both stalk biogenesis and the formation of ectopic cell poles (Fig. 5A) (5, 6). PopZ defines a polar microdomain, recruiting at least 11 other proteins which may additionally include cell wall assembly proteins normally dedicated to stalk biogenesis (7). These growth factors would thus be repositioned to ectopic SpmX/PopZ foci during SpmX overexpression, leading to the formation of new cell poles.

The dependence on PopZ for the outgrowth of ectopic poles reflects similar findings in several other organisms. Notably, in the alphaproteobacterium *Agrobacterium tumefaciens*, which adds new PG at the tip of one cell pole, PopZ colocalizes with a transpeptidase at the growing cell pole, and mislocalization of PopZ causes ectopic growth zones (34, 35). More broadly, homologs of the *Bacillus subtilis* polymeric protein DivIVA, which has many similarities to PopZ, have been linked to polar growth in *Streptomyces coelicolor* and *Mycobacterium smegmatis* through its regulation of PG addition at regions of high membrane curvature (36–39).

### Self-organizing polarity factors.

When overexpressed, SpmX forms ectopic oligomeric aggregates that recruit PopZ to nonpolar sites, opposite from the typical order of assembly. Indeed, PopZ never formed ectopic accumulations without SpmX also being present (Fig. 6C; Fig. S7). The overexpression of SpmX in *Caulobacter* mirrors the coexpression of PopZ and SpmX in *E. coli* in the ability of SpmX to seed additional PopZ foci (Fig. 2B, 4D, and 6C). This phenotype differs from the overexpression of PopZ in *Caulobacter* or in an *E. coli* test system (Fig. 6C), which simply increases the size of existing PopZ regions and is not sufficient to generate new foci (6, 9, 40). Recent work additionally demonstrated that the PopZ binding protein ZitP can also seed a second PopZ focus when coexpressed in *E. coli*, in a manner that requires both PopZ binding and the ZitP transmembrane tether (41). Critically, this similarity between SpmX, ZitP, and other proteins (7) indicates that membrane-bound factors that interact with PopZ may be generally adept at seeding new PopZ microdomains. This potentially widespread mechanism for generation of ectopic microdomains underscores the need for tight spatial and temporal control of polarity factors to prevent cell polarity aberrations.

Our work demonstrates the ordered assembly of the PopZ-SpmX-DivJ signaling complex and that disruption of the relative copy number of the protein components of this complex can drastically alter cell polarity. Ectopic aggregates of SpmX nucleate new poles upon acquisition of colocalized PopZ microdomains, and they specify the identity of those poles based on the recruitment or exclusion of other polar identity proteins. While it is intuitive that self-organizing proteins such as SpmX and PopZ are the most upstream regulators of cell polarity, many questions remain about how these master organizers interact to robustly establish, remodel, and propagate cell polarity through many generations.

## MATERIALS AND METHODS

### Bacterial strains and growth.

For detailed information on strains, plasmids, and growth conditions, consult the supplemental material (see the supplemental methods and tables in Text S1). All *C. crescentus* strains used in this study are derived from the synchronizable wild-type strain CB15N (42) and were grown at 28°C in M2G medium (43). Plasmids used were constructed via Gibson assembly or described in previous studies (45–49).

### Microscopy.

*C. crescentus* and *E. coli* strains were imaged on M2G-1.5% agarose pads. Phase-contrast and fluorescence microscopy images were obtained using a Leica DM6000 B microscope with an HCX PL APO 100×/1.40 oil PH3 CS objective, Hamamatsu electron-multiplying charge-coupled device (EMCCD) C9100 camera, and MetaMorph microscopy automation and image analysis software. For all image panels, the brightness and contrast of the images were balanced with ImageJ (NIH) or Adobe Photoshop CS6 to represent foci and diffuse fluorescent signal.

For computational image analyses, MicrobeTracker (44) was used to determine cell outlines from phase images. Fluorescent signal was integrated along the length of the cell outlines. For fluorescence profiles, a Matlab (MathWorks) script was used to interpolate the integrated fluorescent signal into 50 segments along the cell length. These profiles were averaged for 46 to 210 cells for each experiment. The averaged profiles were normalized such that the highest signal intensity was equal to 1. For *E. coli* cells displaying only diffuse localization, position 0 was randomly set to one cell pole. For *E. coli* cells displaying a fluorescent polar focus, position 0 was set to the cell pole containing the focus. For *Caulobacter* cells, position 0 was set to the stalked pole as visualized via phase images. If no stalk was visible, the pole containing the most intense SpmX-eYFP focus was set at position 0. For all experiments, only cells containing fluorescent signal were analyzed.

### Negative-stain electron microscopy.

Mid-log-phase cultures were applied to glow-discharged carbon-coated grids, stained with 1.5% uranyl acetate, blotted, and air dried. Images were taken at 80 kV on a JEOL TEM1230 transmission electron microscope equipped with a Gatan 967 slow-scan, cooled CCD camera.

### MST binding assays.

SpmX variants were fluorescently labeled on lysine residues with *N*-hydroxysuccinimide-functionalized Atto-488 (Sigma-Aldrich) at approximately one dye molecule per protein. Direct binding between fluorescently labeled SpmX variants and the SpmX targets PopZ, SpmX, and DivJ was probed in 2-fold serial dilutions via microscale thermophoresis (MST) (13, 14) (NanoTemper Technologies). Data analysis was performed as described previously (13). Detailed methods can be found in the supplemental material. Protein purification was performed as described in detail previously (50) and in the supplemental material.

### Size exclusion chromatography.

SpmX variants were assayed for the apparent molecular weight of their assemblies by size exclusion chromatography, using a GE Healthcare Superdex 200 Increase 10/300 GL, connected to a Bio-Rad NGC chromatography system. Molecular weights of the complexes were assigned based on a standard curve derived from the elution volumes of a Bio-Rad premixed gel filtration standard (catalog no. 151-1901). For detailed assay conditions, consult the supplemental material.

### Native gel protein assembly assays.

SpmX variants were assayed for their ability to form higher-order assemblies using nondenaturing gel electrophoresis. Three micrograms of SpmX variant was loaded into each well of a TGX gel (4 to 15%; Bio-Rad), and SpmX complexes were separated by gel electrophoresis at 80 V, for at least 2.5 h, at 4°C. Gels were stained for protein using SafeStain (Invitrogen). Approximations of molecular weight were made using a NativeMark (Thermo Fisher) ladder. Detailed procedures can be found in the supplemental material.
