# Supplementary figures and images for: A Localized Complex of Two Protein Oligomers Controls the Orientation of Cell Polarity
Source: mBio. 2017 Feb 28;8(1):e02238-16. doi: 10.1128/mBio.02238-16 (PMC5347347; doi:10.1128/mBio.02238-16)

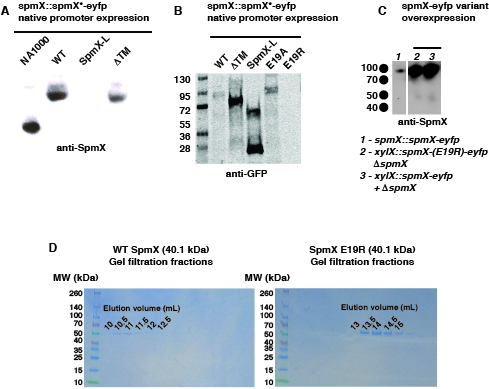

Supplement: FIG S4 [file mbo001173216sf4.tif]
