# Supplementary material for: A Localized Complex of Two Protein Oligomers Controls the Orientation of Cell Polarity
Source: mBio. 2017 Feb 28;8(1):e02238-16. doi: 10.1128/mBio.02238-16 (PMC5347347; doi:10.1128/mBio.02238-16)
Supplement: TEXT S1 [file mbo001173216s1.docx]

**Supplemental Text.**

**Supplemental Discussion**

***Contribution of individual domains to SpmX function* in vivo**

Strains of *Caulobacter* with chromosomal *spmX*-*ΔTM* as the only copy of spmX displayed bipolar accumulation when compared to the full-length protein (59% to 18%, respectively). Several models could explain the role of the TM domains in stalked pole specific accumulation of SpmX. First, we observed that the transmembrane domains of SpmX are required for polar localization of DivJ *in vivo*, suggesting that SpmX may be sequestered within the PopZ matrix away from the membrane, and thus not be positioned to interact with and recruit membrane bound DivJ (Supplemental Fig. S3B, S3C). Second, interaction with DivJ at the stalked pole may secure SpmX in a monopolar position prior to the establishment of the second focus of PopZ at the opposite pole, while the lack of a polar DivJ interaction may consequently result in SpmX associating evenly with the two polar PopZ accumulations. Third, there may be an additional stalked pole-specific factor that promotes monopolar SpmX accumulation in a SpmX TM domain dependent manner. The bactofilins BacA and BacB are attractive candidates for this role, as they also localize to the stalked pole during G1-S transition, where they promote stalk biogenesis and the localization of the peptidoglycan synthase PbpC (1).

It has been shown that the PopZ microdomain contains a space filling polymeric matrix, that extends up to a few hundred nanometers in diameter, suggesting that only a small fraction of PopZ molecules are in direct contact with the cell membrane. The deletion of the SpmX membrane tether, as in SpmX-L and SpmX-ΔTM, would free those SpmX variants to interact with the many PopZ molecules that are not close to the membrane. The failure of these two SpmX variants to recruit DivJ to the stalked pole, despite their own polar localization, suggests that they are binding PopZ molecules distal from the membrane-bound DivJ (Supplemental Fig. S3C). Reciprocally, the broken interaction between DivJ and the SpmX variants may now permit the SpmX variants to interact with PopZ at either pole, rather than in the stalked-pole specific WT PopZ-SpmX-DivJ complex (Supplemental Fig. S3C).

***An alternative model for ectopic pole generation***

An alternative version of the ‘recruitment hypothesis’ is that SpmX directly recruits factors involved in cell wall growth that are membrane-bound or in the periplasm, independently of PopZ, explaining the strict requirement of the TM domain for ectopic pole formation (Fig. 6C). In this model, the apparent contribution of PopZ to ectopic pole formation may instead be due to its role in ordering chromosome segregation via its known interaction with the Par A and ParB segregation factors (2). Specifically, without PopZ to direct the Par proteins and with multiple cells poles that a segregating chromosome could move toward, there could be a synthetic lethal defect in chromosome segregation specific to a multi-pole cell body shape.

**Supplemental Materials and Methods**

**Plasmid construction**

pAP139 was constructed by amplifying mCherry-PopZ from pGB528 (3) with engineered 5’ NcoI and 3’ EcoRI sites. Both the PCR product and pBad/HisA were digested with NcoI and EcoRI, and the PCR was inserted into the digested vector with T4 DNA ligase (NEB). pAP371 was constructed via Gibson assembly (4). SpmX was amplified from chromosomal *Caulobacter* DNA and eYFP was amplified from pYFPC-4. The PCR products were amplified with oligonucleotides containing 15 base pairs of homology to the digested vector and to the adjacent insert DNA pieces. A linker (GGSGS) between the SpmX and eYFP coding sequences was encoded into the oligonucleotides. The DNA pieces were assembled into pACYC-1 digested with NdeI. pAP372 was assembled in a similar manner to pAP371, via assembly of DivJ and eCFP into NcoI digested pACYC-1. pAP381 was constructed by Gibson assembly of DivJ-eCFP amplified from pAP381 into NcoI digested pAP371.

To generate pAP376 and pAP377, inverse PCR with 5’ phosphorylated oligonucleotides was performed on pAP371. The PCR products were designed to delete the indicated residues and were ligated with T4 DNA ligase. pAP423 was created by amplifying SpmX Δ156-431 from pAP376, and assembly into pAP372 digested with NdeI via Gibson assembly.

pAP411 was constructed by amplifying 941 base pairs upstream of the SpmX coding sequence together with the SpmX ORF from *Caulobacter* genomic DNA. This PCR product was inserted into pYFPC-2 digested with NdeI via Gibson assembly. pAP476 and pAP477 were created via inverse PCR with 5’ phosphorylated oligonucleotides using pAP411 as a template. The PCR products were designed to delete the indicated residues and were ligated with T4 DNA ligase. pAP482, pAP483, pAP484, and pAP485 were created via Quickchange mutagenesis of pAP411. pAP503 was created via Quickchange mutagenesis of pAP484.

To generate pAP549, SpmX was amplified and inserted into pXCFPC-2 digested with NdeI and NheI (removing the eCFP coding sequence) via Gibson assembly. To generate pAP537, pAP557, pAP588, and pAP589, SpmX variants were amplified off of pAP483, pAP411, pAP476, and pAP477, respectively. These PCR products were inserted into pXYFPC-2 digested with NdeI and EcoRI via Gibson assembly. To generate pAP596, the 941 base pair sequence upstream of SpmX was amplified together with SpmX-eYFP off of pAP411. This PCR product was inserted into pBXMCS-6 digested with BamHI and NdeI (which removes the xylose promoter within the plasmid) via Gibson assembly. pAMP1 was constructed via amplifying 700 base pairs of genomic sequence upstream of the PopZ coding sequence and mCherry-PopZ. These PCR products were inserted into pNTPS138 digested with SpeI and EcoRI via Gibson assembly. To construct pAP496, 500 base pairs of genomic sequence upstream and downstream of the DivJ coding sequence were amplified and inserted into pNTPS138 digested with EcoRI and AflII via Gibson assembly.

For all Gibson assembly reactions, inserts were generated via PCR with 15 base pairs of homology to adjacent assembly pieces.

***C. crescentus* strains and cell growth**

All *C. crescentus* strains used in this study are derived from the synchronizeable wild-type strain CB15N (5) and were grown at 28°C in M2G medium (6). Generalized transduction was performed with phage ϕCr30 as described (6). When appropriate, growth media was supplemented with the following antibitoics: 0.5 μg/mL gentamicin, 5 μg/mL kanamycin, 1 μg/mL oxytetracyclin, 25 μg/mL spectinomycin, and/or 1 μg/mL chloramphenicol. Expression of proteins was stimulated by the addition of vanillate (pH 7.5) or _D_-xylose at the indicated concentrations and for the indicated time periods before imaging. Strains were analyzed at mid-exponential phase of growth for imaging. Descriptions of strains and plasmids used in this study are in Supplementary Tables 1 and 2.

**Heterologous expression of fluorescent fusions in *E. coli***

mCherry-PopZ was placed under the control of an arabinose inducible promoter on the pBad vector (Thermo Fisher Scientific). SpmX-eYFP or DivJ-eCFP were placed under the control of one of two T7 polymerase promoters on the pACYC-Duet vector (EMD Millipore). These two plasmids contain compatible origins of replication. The pBad::mCherry-PopZ and either the pACYC::SpmX-eYFP or pACYC::DivJ-eCFP plasmids were placed into the *E. coli* BL21(DE3) strain. Strains were grown at 37 ^o^C in LB supplemented with ampicillin (pBad) and chloramphenicol (pAYC-Duet) to an OD_600_=0.3. Cells were induced with 0.2% D-arabinose (pBad::mCherry-PopZ), 100 μM IPTG (pACYC::SpmX-eYFP/DivJ-eCFP), or both for 1 hour at 37 ^o^C before imaging.

To assay the co-localization of all three proteins in *E. coli,* a pACYC-Duet plasmid was constructed with SpmX-eYFP and DivJ-eCFP under control of both T7 polymerase promoters on the pACYC-Duet vector. This plasmid was co-transformed with the pBad::mCherry-PopZ plasmid into *E. coli*, and culturing/expression was performed as described above.

The localization of SpmX-eYFP variants were assayed in *E. coli* in a similar manner, with the wild-type SpmX sequence replaced with the indicated mutant allele.

**Overexpression of SpmX variants**

To overexpress SpmX, the SpmX coding sequence or a SpmX-eCFP fusion was placed under control of a xylose inducible promoter and integrated at the native *xylX* chromosomal locus in an otherwise wild-type background, or in *ΔpopZ, ΔdivJ,* or *ΔtipN* strains. Cells were grown in M2G with the appropriate selective antibiotic (kanamycin or chloramphenicol) to the mid-log phase of exponential growth. The cultures were back diluted into fresh M2G media supplemented with 0.3% xylose and antibiotic and grown for 18 hours. To visualize FtsZ-eYFP during SpmX-eCFP overexpression, FtsZ-eYFP was placed under control of a vanillate inducible promoter at the native *vanA* chromosomal locus in the SpmX-eCFP overexpression strain described above. SpmX-eCFP was overexpressed as described, with the addition of FtsZ-eYFP induction via 50 μM vanillate for 30 minutes prior to imaging.

To assay the ability of SpmX variants to generate ectopic poles, SpmX-eYFP and corresponding eYFP fusions to SpmX variants were placed under control of the xylose locus at the chromosomal *xylX* locus in a *ΔspmX* strain. Overexpression of SpmX was performed as described above, and generated a similar number of cells displaying ectopic poles as when SpmX was overexpressed in a wild-type background. To overexpress SpmX-eYFP from its native promoter, SpmX-eYFP plus 1 kB of genomic sequence upstream of SpmX were cloned into a high-copy vector (pBXMCS-6). This plasmid was transformed into a *ΔspmX* strain.

**Surface plasmon resonance binding assays**

Surface plasmon resonance experiments were performed on a BIACORE 3000 biosensor system (GE Healthcare) at 25ºC. PopZ was covalently immobilized on the surface of a CM5 biosensor chip (GE Healthcare) by amine coupling chemistry using *N*-hydroxysuccinimide (NHS) and *N*′-(3-dimethylaminopropyl) carbodiimide hydrochloride (EDC) according to the manufacturer’s instructions. To investigate binding of SpmX or SpmX-L to the immobilized PopZ, dialyzed SpmX or SpmX-L, the proteins were dialyzed against Kinase Buffer (50mM Hepes, pH 8, 200mM KCl, 2mM MgCl2, 1mM DTT), diluted in Kinase Buffer, and injected over the PopZ surface for 2.5 minutes at different concentrations at a flow rate of 30μl/min. For each experiment at least 5 different concentrations of SpmX or SpmX-L were injected over each experimental and control flow cell. Dissociation was allowed to occur at the same flow rate for 300 sec. followed by Running Buffer alone at a flow rate of 100μl/min to allow the baseline to stabilize. All data were corrected for unspecific binding by subtracting the signal measured in a control cell lacking immobilized ligand. Data analysis was performed using the BIAevaluation software 4.1 (GE Healthcare).

**Immunoblots**

*Caulobacter* cells were harvested at mid-log phase, lysed in Laemmli buffer, and subjected to SDS-PAGE. For panels A and C, the proteins were transferred to a PVDF membrane and blotted with anti-SpmX antibody (1:50,000) (7), followed by a goat-anti-rabbit secondary antibody conjugated to horseradish peroxidase. For panel B, the proteins were transferred to a nitrocellulose protein, and blotted with anti-GFP antibody (1:2,000; Cell Signaling), followed by goat-anti-rabbit secondary antibody conjugated to an infrared fluorophore, and imaged with an Odyssey infrared imaging system (Li-Cor). Where applicable, SpmX variants were induced with 0.3% xylose for 18 hours prior to lysis.

**Protein purification**

PopZ, WT SpmX (residues1-356), SpmX-L (residues 1-155), and SpmX E19R (residues 1-356), and DivJ (residues 188-585), were expressed and purified from E. coli. For each protein, either BL21 (SpmX variants, DivJ) or Rosetta (PopZ) strains of E. coli were grown to an OD of 0.4, and then switched to induction temperatures of 28^o^C (SpmX, DivJ) or 30^o^C (PopZ) for half an hour prior to induction. Expression was induced with 1 mM IPTG for 2 hours (PopZ), or with 500 μM IPTG for 3 hours (SpmX, DivJ). Cell pellets were collected via centrifugation and stored at -80^o^C.

PopZ was purified under denaturing conditions similarly to a previous description (3). The frozen cell pellet was resuspended in lysis buffer containing 100 mM Na-phosphate, 10 mM Tris-Cl pH 8.0, 300 mM NaCl, 8 M urea, 20 mM imidazole, and 1 tablet of EDTA-free UltraCruz protease inhibitors (Santa Cruz Biotechnology) for each 50 mL of lysis buffer. 1.2 g of guanidinium chloride was added per 1L cell culture, and the cells were homogenized by passage through a 21-gauge needle. Insoluble material was removed via centrifugation at 12,100 g for 45 minutes at room temperature. The supernatant was incubated for 2 hours with 1 mL His-Pur Ni-NTA agarose resin (ThermoFisher) per 1 L of initial cell culture. The resin was washed 4 times in lysis buffer. The protein was eluted using 4 mL elution buffer (lysis buffer modified to have 250 mM imidazole) per 1 L initial culture. The protein was concentrated using a 30 k MWCO spin filter (Millipore). The protein was refolded during 3 rounds of dialysis at 4^o^C in 20 mM Tris-Cl buffer at pH 8.0.

SpmX variants and DivJ were purified similarly to a previous description (8). The cell pellets were resuspended in lysis buffer containing 500 mM KCl, 50 mM HEPES-KOH pH 8.0, 10% glycerol, 1 mM DTT, 25 mM imidazole, 0.3 μL benzonase nuclease per 1 L initial culture, and 1 tablet of EDTA-free UltraCruz protease inhibitors (Santa Cruz Biotechnology) per 50 mL lysis buffer. Cells were lysed via three passes through an Emulsiflex (Avestin), and insoluble material was removed via centrifugation at 29,000 g for 45 minutes at 4^o^C. The supernatant was collected and incubated with 1 mL His-Pur NTA agarose resin (ThermoFisher) per 1 L initial cell culture. The resin was washed four times in lysis buffer, and the protein was eluted using 3 mL elution buffer (lysis buffer modified to have 250 mM imidazole) per 1 L initial culture. The proteins were concentrated and dialyzed into storage buffer containing 200 mM KCl, 50 mM HEPES-KOH pH 8.0, 10% glycerol, and 1 mM DTT. The proteins were flash frozen and stored at -80^o^C.

**Lysozyme activity assay**
*Micrococcus lysodeikticus*lyophilized cells (Sigma-Aldrich) were resuspended in 50 mM HEPES pH 8.0, 100 mM NaCl to an OD450=0.6. 200 μL of the suspension were dispensed into wells of a 96-well plate.  Hen egg white lysozyme (Sigma-Aldrich) or SpmX (1-355) were added to the suspension to a final concentration of 20 μM in final volume of 250 μL. For negative controls, 50 μL of buffer was added to the lyophilized cell suspension. The reactions were mixed by pipetting and the absorbance at 450 nm was measured every minute for 30 minutes at room temperature in a Molecular Devices Spectra Max M5 plate reader.

**Microscale thermophoresis (MST) binding assays**

Fluorescent labeling of lysine residues in SpmX WT (residues 1-356), SpmX-L (residues 1-155), and SpmX E19R was accomplished by incubating each protein with an N-hydroxysuccinimide (NHS) ester conjugated to Atto-488 (Sigma-Aldrich). The dye conjugate was dissolved in dry DMSO to make a 1 mM solution. The conjugation reaction was performed in the dark using 1-2 mg/mL protein and a 3-fold molar ratio of dye to protein at room temperature, with gentle shaking. Unconjugated dye was removed using a single pass through a PD SpinTrap G-25 (GE Healthcare) spin column. The labeled protein was diluted to 2-4 μM and flash frozen in storage buffer containing 200 mM KCl, 50 mM HEPES-KOH pH 8.0, 10% glycerol, and 1 mM DTT.

Direct binding between fluorescently labeled SpmX variants and the SpmX targets PopZ, SpmX, and DivJ was probed via microscale thermophoresis (9, 10) (NanoTemper Technologies). For each binding experiment, a twofold serial dilution was made for each protein target in storage buffer (lacking the DTT, with 0.025% Tween-20). Fluorescently labeled SpmX was then added at 25 nM (WT and SpmX-L) or 50 nM (E19R), mixed, and incubated at room temperature for 10 minutes, covered, in the dark. The protein mixtures were loaded into Standard Treated capillaries (NanoTemper). Binding was assessed using the following instrument settings: 70% blue LED power, 40% IR-laser power, 30 second IR heating period, 5 second recovery.

Binding data were initially fit in MO.Affinity Analysis (NanoTemper), and the binding curve plateau data were exported. Experimental replicates were averaged in Prism 7 (GraphPad) and according to the law of mass action, as described (10):

$$\frac{BL}{B_{0}}=\frac{\left( \left[ L_{0} \right]+\left[ B_{0} \right]+K_{d} \right)-\sqrt{((\left[ L_{0} \right]+\left[ B_{0} \right]+K_{d})^{2}-4*\left[ L_{0} \right]*\left[ B_{0} \right])}}{2[B_{0}]}$$

In this equation, BL represents the concentration of protein complexes, [B_0_] represents total binding sites of the fluorescent ligand, [L_0_] represents the amount of added ligand, and K_d_ represents the dissociation constant.

**Size exclusion chromatography**

SpmX variants were assayed for the apparent molecular weight of their assemblies by size exclusion chromatography. Purified samples of WT SpmX (1-355), SpmX E19R (1-355), and SpmX-L (1-155) were injected as 500 μL volumes at approximately 20 μM each onto a GE Healthcare Superdex 200 Increase 10/300 GL, connected to a BioRad NGC Chromatography System. Samples were passed through the column at 0.5 mL/min in buffer containing 200 mM KCl and 50 mM HEPES-KOH pH 8.0, and protein elution was detected by measuring light absorbance at 215 nm. Protein content in the eluted fractions was re-verified by SDS-PAGE analysis. Molecular weights of the complexes were assigned based on a standard curve derived from the elution volumes of a BioRad premixed gel filtration standard (cat #151-1901). A linear baseline adjustment was applied to correct for detector drift during the WT SpmX and SpmX-L separations. A representative trace is shown for each construct from two independent repetitions of the separation.

**Native gel protein assembly assays**

SpmX variants were assayed for their ability to form higher order assemblies using non-denaturing gel electrophoresis. 3 μg of SpmX variant was loaded into each well of a TGX gel (4-15%, Bio-Rad), and SpmX complexes were separated by gel electrophoresis at 80 V, for at least 2.5 hours, at 4^o^C. SpmX gel loading buffer contained 200 mM KCl, 50 mM HEPES-KOH pH 8.0, 20% glycerol, and the gel was run in Tris-glycine buffer containing no denaturant. For refolding experiments, the SpmX variants were first denatured for 30 minutes at room temperature, by diluting the proteins fivefold in a denaturing buffer containing 200 mM KCl, 50 mM HEPES-KOH pH 8.0, 10% glycerol, and 8 M urea (final volume 100 μL). The proteins were then refolded overnight at 4^o^C via dialysis in 1 L of buffer lacking the urea. For co-oligomerization experiments, the proteins were mixed at the listed molar ratios, and incubated together at room temperature for 15 minutes, prior to loading 2-4 μg of protein per well in the gel. Gels were stained for protein using SafeStain (Invitrogen). Approximations of molecular weight were made using a NativeMark (ThermoFisher) ladder.

**Supplemental Tables**

**Table S1: Bacterial Strains**

| ***C. crescentus* strains** | **Relevant genotype/sescription** | **Construction, source or reference** |
| --- | --- | --- |
| AP248 | *xylX::SpmX-eCFP* | pAP206 electroporated into CB15N |
| AP251 | *xylX::SpmX-eCFP; popZ::mCherry-PopZ* | pAP206 electroporated into cAMP1 and counterselected |
| AP400 | *spmX::SpmX-eYFP E19R* | pAP483 electroporated into *ΔspmX* |
| AP401 | *spmX::SpmX-eYFP D30A* | pAP484 electroporated into *ΔspmX* |
| AP414 | *spmX::SpmX-eYFP* | pAP411 electroporated into *ΔspmX* |
| AP443 | *spmX::SpmX-eYFP Δ356-431* | pAP477 electroporated into *ΔspmX* |
| AP486 | *spmX::SpmX-eYFP Δ156-431* | pAP476 electroporated into *ΔspmX* |
| AP488 | *xylX::SpmX-eCFP; tipN::TipN-mCherry* | AP248 transduced into AP558 |
| AP490 | *spmX::SpmX-eYFP E19A* | pAP482 electroporated into *ΔspmX* |
| AP491 | *spmX::SpmX-eYFP D30A* | pAP485 electroporated into *ΔspmX* |
| AP496 | *divJ::Δ* | pAP496 electroporated into CB15N and counterselected |
| AP504 | *spmX::SpmX-eYFP E19A D30A* | pAP503 electroporated into *ΔspmX* |
| AP531 | *xylX::SpmX-eCFP; popZ::mCherry-PopZ* | JP369 transduced into AP248 |
| AP546 | *xylX::SpmX-eCFP; popZ::Δ* | AP248 transduced into GB255 |
| AP547 | *xylX::SpmX-eCFP; divJ::Δ* | AP248 transduced into AP497 |
| AP550 | *xylX::SpmX* | AP549 electroporated into CB15N |
| AP555 | *xylX::SpmX-eCFP; vanA::FtsZ-eYFP* | EG490 kan marker transduced into AP248 |
| AP558 | *tipN::TipN-mCherry* | pEG471 electroporated into CB15N |
| AP564 | *xylX::SpmX-eCFP; tipN::Δ* | AP557 electroporated into *ΔtipN* |
| AP569 | *spmX::SpmX-eYFP; divJ::DivJ-mCherry* | AP414 transduced into GB212 |
| AP570 | *spmX::SpmX-eYFP Δ356-431; divJ::DivJ-mCherry* | AP443 transduced into GB212 |
| AP572 | *spmX::SpmX-eYFP Δ156-431; divJ::DivJ-mCherry* | AP486 transduced into GB212 |
| AP575 | *xylX::SpmX-eYFP; divJ::DivJ-mCherry* | AP248 transduced into GB212 |
| cAMP1 | *popZ::mCherry-PopZ* | pAMP1 electroporated into CB15N and counterselected |
| THM189 | *xylX::SpmX-eYFP; spmX::Δ* | pAP557 electroporated into *ΔspmX* |
| THM188 | *xylX::SpmX E19R-eYFP; spmX::Δ* | pAP537 electroporated into *ΔspmX* |
| THM190 | *xylX::SpmX Δ156−431-eYFP; spmX::Δ* | pAP588 electroporated into *ΔspmX* |
| THM191 | *xylX::SpmX Δ356−431-eYFP; spmX::Δ* | pAP589 electroporated into *ΔspmX* |
| THM192 | *PspmX::spmX-eYFP* high copy plasmid*; spmX::Δ* | pAP596 electroporated into *ΔspmX* |
| CB15N | Synchronizeable derivative of WT CB15 | (11) |
| EG490 | *mipZ::mipZ–cerulean vanA::PvanA–ftsZ–yfp* | (12) |
| GB212 | *divJ::DivJ-mCherry* | (13) |
| GB255 | *popZ::Δ* | (14) |
| JP369 | *popZ::mCherry-PopZ* | (2) |
| MT190 | *parB::eCFP-ParB* | (15) |
| *ΔSpmX* | *spmX::Δ* | (7) |
| *ΔTipN* | *tipN::Δ* | (16) |
| ***E. coli* stains** | **Relevant genotype/description** | **Source** |
| BL21(DE3) | Protein expression | NEB |
| AP201 | *pBad::mCherry-PopZ* | pAP139 transformed into BL21 |
| AP202 | *pBad::mCherry-PopZ R3* | pGB786 transformed into BL21 |
| AP373 | *pBad::mCherry-PopZ; pT7::SpmX-eYFP* | pAP371 transformed into AP201 |
| AP382 | *pBad::mCherry-PopZ; pT7::SpmX-eYFP, DivJ-eCFP* | pAP381 transformed into AP201 |
| AP384 | BL21; *pBad::mCherry-PopZ; pT7::SpmX-eYFP*  *Δ156-431* | pAP376 transformed into AP201 |
| AP385 | *pBad::mCherry-PopZ; pT7::SpmX-eYFP*  *Δ1-155* | pAP377 transformed into AP201 |
| AP389 | BL21; *pBad::mCherry-PopZ; pT7::DivJ-eCFP* | pAP372 transformed into AP201 |
| AP418 | *pBad::mCherry-PopZ R3; pT7::SpmX-eYFP* | pAP371 transformed into AP202 |
| AP426 | *pBad::mCherry-PopZ; pT7::SpmX-eYFP Δ156-431, DivJ-eCFP* | pAP424 transformed into AP201 |
| AP434 | *pTev + 6His-DivJ Δ1-187* | DivJ cloned into pTev cut w/ NheI |
| AP435 | *pET28b + SpmX-6His Δ356-431* | SpmX cloned into 5’ NcoI and 3’ EcoRI restriction sites |
| AP438 | *pET28b + SpmX-6His Δ156-431* | SpmX cloned into 5’ NcoI and 3’ EcoRI restriction sites |
| AP538 | *pET28b + SpmX-6His Δ356-431 (E19R)* | SpmX cloned into 5’ NcoI and 3’ EcoRI restriction sites |
|  |  |  |
| BL21 Rosetta | Protein expression | Novagen |
| GB169 | *pET28a + 6His-PopZ* | (14) |

**Table S2: Plasmids**

| **Plasmid** | **Description** | **Backbone** | **Source** |
| --- | --- | --- | --- |
| pBXMCS-6 | High copy replicating plasmid | pBXMCS-6 | (17) |
| pXCFPC-6 | Integrating plasmid | pXCFPC-6 | (17) |
| pXCFPC-2 | Integrating plasmid | pXCFPC-2 | (17) |
| pXYFPC-2 | Integrating plasmid | pXYFPC-2 | (17) |
| pYFPC-4 | Integrating plasmid | pYFPC-4 | (17) |
| pVCHYC-4 | Integrating plasmid | pVCHYC-4 | (17) |
| pEG471 | *PtipN::TipN-mCherry* integrates at *tipN* locus | pVCHYC-4 | (12) |
| pNPTS138 | Intergrating plasmid containing selection for double recombination | pNPTS138 | M.R.K Alley |
| pGB787 | *Pbad::mCherry-PopZ R3* | pBad/HisA | Grant Bowman |
| pAP139 | *Pbad::mCherry-PopZ* | pBad/HisA | This study |
| pAP140 | *Pbad::mCherry-PopZ R3* | pBad/HisA | This study |
| pAP206 | *Pxyl::SpmX-eCFP* integrates at *xylX* locus | pXCFPC-6 | Jerod Ptacin |
| pAP371 | *PT7::SpmX-eYFP* | pACYCDuet-1 | This study |
| pAP372 | *PT7::DivJ-eCFP* | pACYCDuet-1 | This study |
| pAP376 | *PT7::SpmX-eYFP Δ156-431* | pACYCDuet-1 | This study |
| pAP377 | *PT7::SpmX-eYFP Δ1-155* | pACYCDuet-1 | This study |
| pAP381 | *PT7::SpmX-eYFP; PT7::DivJ-eCFP* | pACYCDuet-1 | This study |
| pAP411 | *Pspmx::SpmX-eYFP* integrates at *spmX* locus | pYFPC-4 | This study |
| pAP423 | *PT7::SpmX-eYFP Δ156-431; PT7::DivJ-eCFP* | pACYCDuet-1 | This study |
| pAP476 | *Pspmx::SpmX-eYFP Δ156-431* integrates at *spmX* locus | pYFPC-4 | This study |
| pAP477 | *Pspmx::SpmX-eYFP Δ356-431* integrates at *spmX* locus | pYFPC-4 | This study |
| pAP482 | *Pspmx::SpmX-eYFP E19A* integrates at *spmX* locus | pYFPC-4 | This study |
| pAP483 | *Pspmx::SpmX-eYFP E19R* integrates at *spmX* locus | pYFPC-4 | This study |
| pAP484 | *Pspmx::SpmX-eYFP D30A* integrates at *spmX* locus | pYFPC-4 | This study |
| pAP485 | *Pspmx::SpmX-eYFP D30R* integrates at *spmX* locus | pYFPC-4 | This study |
| pAP503 | *Pspmx::SpmX-eYFP E19A D30R* integrates at *spmX* locus | pYFPC-4 | This study |
| pAP549 | *Pxyl::SpmX* integrates at *xylX* locus | Inserted into pXCFPC-2 at 5’ NdeI and 3’ NheI sites to remove eCFP | This study |
| pAP537 | *Pxyl::spmX E19R-eYFP* integrates at *xylX* locus | pXYFPC-2 | This study |
| pAP557 | *Pxyl::spmX-eYFP*  integrates at *xylX* locus | pXYFPC-2 | This study |
| pAP588 | *Pxyl::spmX Δ156-431* integrates at *xylX* locus | pXYFPC-2 | This study |
| pAP589 | *Pxyl::spmX Δ356-431* integrates at *xylX* locus | pXYFPC-2 | This study |
| pAP596 | *PspmX::spmX-eYFP* on a high copy plasmid | pBXMCS-6 | This study |
| pAMP1 | *PpopZ::mCherry-PopZ* integrates at *popZ* locus | pNPTS138 | This study |
|  |  |  |  |

**Supplemental References**

1. **Kühn J**, **Briegel A**, **Mörschel E**, **Kahnt J**, **Leser K**, **Wick S**, **Jensen GJ**, **Thanbichler M**. 2010. Bactofilins, a ubiquitous class of cytoskeletal proteins mediating polar localization of a cell wall synthase in Caulobacter crescentus. EMBO J **29**:327–39.

2. **Ptacin JL**, **Gahlmann A**, **Bowman GR**, **Perez AM**, **von Diezmann ARS**, **Eckart MR**, **Moerner WE**, **Shapiro L**. 2014. Bacterial scaffold directs pole-specific centromere segregation. Proc Natl Acad Sci U S A **111**:E2046-55.

3. **Bowman GR**, **Perez AM**, **Ptacin JL**, **Ighodaro E**, **Folta-Stogniew E**, **Comolli LR**, **Shapiro L**. 2013. Oligomerization and higher-order assembly contribute to sub-cellular localization of a bacterial scaffold. Mol Microbiol 1–20.

4. **Gibson DG**, **Young L**, **Chuang R-Y**, **Venter JC**, **Hutchison CA**, **Smith HO**. 2009. Enzymatic assembly of DNA molecules up to several hundred kilobases. Nat Methods **6**:343–5.

5. **Poindexter JS**. 1964. Biological properties and classification of the Caulobacter group. Bacteriol Rev **28**:231–95.

6. **Ely B**. 1991. Genetics of Caulobacter crescentus. Methods Enzymol **204**:372–384.

7. **Radhakrishnan SK**, **Thanbichler M**, **Viollier PH**. 2008. The dynamic interplay between a cell fate determinant and a lysozyme homolog drives the asymmetric division cycle of Caulobacter crescentus. Genes Dev **22**:212–225.

8. **Mann TH**, **Childers WS**, **Blair J a**, **Eckart MR**, **Shapiro L**. 2016. A cell cycle kinase with tandem sensory PAS domains integrates cell fate cues. Nat Commun **7**.

9. **Seidel SAI**, **Dijkman PM**, **Lea WA**, **van den Bogaart G**, **Jerabek-Willemsen M**, **Lazic A**, **Joseph JS**, **Srinivasan P**, **Baaske P**, **Simeonov A**, **Katritch I**, **Melo FA**, **Ladbury JE**, **Schreiber G**, **Watts A**, **Braun D**, **Duhr S**. 2013. Microscale thermophoresis quantifies biomolecular interactions under previously challenging conditions. Methods **59**:301–315.

10. **Wienken CJ**, **Baaske P**, **Rothbauer U**, **Braun D**, **Duhr S**. 2010. Protein-binding assays in biological liquids using microscale thermophoresis. Nat Commun **1**:100.

11. **Evinger M**, **Agabian N**. 1977. Envelope-Associated Nucleoid from Caulobacter crescentus Stalked and Swarmer Cells. J Bacteriol **132**:294–301.

12. **Goley ED**, **Yeh Y-C**, **Hong S-H**, **Fero MJ**, **Abeliuk E**, **McAdams HH**, **Shapiro L**. 2011. Assembly of the Caulobacter cell division machine. Mol Microbiol **80**:1680–1698.

13. **Bowman GR**, **Comolli LR**, **Gaietta GM**, **Fero M**, **Hong S-H**, **Jones Y**, **Lee JH**, **Downing KH**, **Ellisman MH**, **McAdams HH**, **Shapiro L**. 2010. Caulobacter PopZ forms a polar subdomain dictating sequential changes in pole composition and function. Mol Microbiol **76**:173–89.

14. **Bowman GR**, **Comolli LR**, **Zhu J**, **Eckart M**, **Koenig M**, **Downing KH**, **Moerner WE**, **Earnest T**, **Shapiro L**. 2008. A Polymeric Protein Anchors the Chromosomal Origin/ParB Complex at a Bacterial Cell Pole. Cell **134**:945–955.

15. **Thanbichler M**, **Shapiro L**. 2006. MipZ, a Spatial Regulator Coordinating Chromosome Segregation with Cell Division in Caulobacter. Cell **126**:147–162.

16. **Huitema E**, **Pritchard S**, **Matteson D**, **Radhakrishnan SK**, **Viollier PH**. 2006. Bacterial birth scar proteins mark future flagellum assembly site. Cell **124**:1025–1037.

17. **Thanbichler M**, **Iniesta A a**, **Shapiro L**. 2007. A comprehensive set of plasmids for vanillate- and xylose-inducible gene expression in Caulobacter crescentus. Nucleic Acids Res **35**:e137.
